# Low-Temperature Ethanol Sensor via Defective Multiwalled Carbon Nanotubes

**DOI:** 10.3390/ma15134439

**Published:** 2022-06-23

**Authors:** Nagih M. Shaalan, Faheem Ahmed, Mohamed Rashad, Osama Saber, Shalendra Kumar, Abdullah Aljaafari, Adil Ashoaibi, Amera Z. Mahmoud, Mohammed Ezzeldien

**Affiliations:** 1 Department of Physics, College of Science, King Faisal University, Al-Ahsa 31982, Saudi Arabia; fahmed@kfu.edu.sa (F.A.); osmohamed@kfu.edu.sa (O.S.); sjagdish@kfu.edu.sa (S.K.); aaljaafari@kfu.edu.sa (A.A.); adshoaibi@kfu.edu.sa (A.A.); 2 Physics Department, Faculty of Science, Assiut University, Assiut 71516, Egypt; m.ahmad@ut.edu.sa (M.R.); a.elshahedy@qu.edu.sa (A.Z.M.); 3 Physics Department, Faculty of Science, University of Tabuk, Tabuk 71491, Saudi Arabia; 4 Egyptian Petroleum Research Institute, Nasr City, Cairo 11727, Egypt; 5 Department of Physics, School of Engineering, University of Petroleum & Energy Studies, Dehradun 248007, India; 6 Department of Physics, College of Sciences and Art at ArRass, Qassim University, ArRass 51921, Saudi Arabia; 7 Department of Physics, College of Science, Jouf University, Sakaka 72388, Saudi Arabia; meabas@ju.edu.sa; 8 Metallurgy & Material Science Tests (MMST) Lab., Department of Physics, Faculty of Science, South Valley University, Qena 83523, Egypt

**Keywords:** 1D nanostructures, defective carbon nanotubes, sensing properties, ethanol sensor

## Abstract

This paper focuses on the fabrication of defective-induced nanotubes via the catalytic chemical vapor deposition method and the investigation of their properties toward gas sensing. We have developed defective multi-walled carbon nanotubes with porous and crystalline structures. The catalyst layer used in CNTs’ growth here was based on 18 and 24 nm of Ni, and 5 nm of Cr deposited by the dc-sputtering technique. The CNTs’ defects were characterized by observing the low graphite peak (G-band) and higher defect peaks (D-band) in the Raman spectrum. The defectives sites are the main source of the sensitivity of materials toward different gases. Thus, the current product was used for sensing devices. The device was subjected to various gases such as NO, NO_2_, CO, acetone, and ethanol at a low operating temperature of 30 °C and a concentration of 50 ppm. The sensor was observed to be less sensitive to most gas while showing the highest response towards ethanol gas. The sensor showed the highest response of 8.8% toward ethanol at 30 °C of 50 ppm, and a low response of 2.8% at 5 ppm, which was investigated here. The signal repeatability of the present sensor showed its capability to detect ethanol at much lower concentrations and at very low operating temperatures, resulting in reliability and saving power consumption. The gas sensing mechanism of direct interaction between the gas molecules and nanotube surface was considered the main. We have also proposed a sensing mechanism based on Coulomb dipole interaction for the physical adsorption of gas molecules on the surface.

## 1. Introduction

With the growth of humanity, the demand for the use of technology in all aspects of life increased, including the demand for different gases for use in many fields. Thus, the demand for monitoring these gases increased. Among these gases is ethanol, whose control is in high human demand since it is the basis of alcohol consumed by humans, with 3 million deaths every year worldwide a result of the harmful use of alcohol, representing 5.3% of all deaths [1]. Ethanol is a preservative and antifungal due to its ability to denature the proteins in yeasts and molds. Before sealing food, ethanol can be injected inside the package to create an antimicrobial effect in cases of long-term storage, or it can be done using ethanol generation bags [2,3,4]. Ethanol is also used to prevent the growth of moldy mildew in high-moisture bakery and fish products [4]. However, the main drawback of using ethanol vapor for preservation purposes is its unpleasant odor and undesirable flavor formation in foods. Moreover, the use of ethanol may lead to its absorption in food to significant levels, which may raise concerns. Ethanol is also used as a good biofuel because it does not emit carbon dioxide, unlike gasoline or diesel. Thus, its use as part of a vehicle fuel mixture has many environmental benefits in terms of reducing hazardous emissions in vehicles [5,6,7,8]. Its presence in the form of a liquid or gas also has advantages and makes it attractive in the process of transporting it as a fuel. In addition, it is the most common compound used in many fields, including the scientific, medical, and industrial fields. Ethanol sensors with efficient and discriminating sensitivity are highly used in the testing of packaged food, in the wine industry for fermentation process controlling, in detecting drunken drivers by traffic police, in different medical applications, and many other applications [9,10,11,12]. Because of its growing use, monitoring its vapor during its production process is an important need, along with safety challenges that require accurate detection of its vapor at very small levels.

Gas sensors are one of the most important technologies required when producing or manufacturing such gases. Through this, it is possible to monitor productivity as well as the leakage of gases into the surrounding environment. Therefore, a large number of researchers have paid great attention to the process of creating gas sensors by methods other than those that are expensive techniques such as gas chromatography [13,14], light waves [15,16], and sound waves [17]. The technique for electrochemical sensors and chemical resistance is attractive, such as a gas sensor using metal oxides, graphene, and multilayer carbon tubes [18,19,20,21,22,23,24,25,26,27], as chemical resistance sensors are simple and have the advantage of being integrated into a microsensor. CNT chemical resistance sensors are widely used due to their response to many gases, room temperature operation, extremely low detection feature, and low power consumption [23,25,28]. Therefore, many researchers have used carbon tubes, whether grafted or not, in the manufacture of sensors for different gases. 

The conductivity/chemical resistor measurement sensor has the advantage of forming a miniature sensing system, and carbon nanotubes or metallic nanoparticles have been used as sensors for chemical-resistor-based sensors, which have good conductivity and internal gas sensing properties [29,30]. In previous studies, it was found that the adsorption of gas particles can be affected by defects in the tubes or defects at the ends of the carbon nanotube wall, where the defect sites can provide additional places for the bonding of gas molecules with the tubes [31]. It is reported that graphene reacted with CO, NO, and NO_2_ when it was doped with defective elements [32,33,34]. The study showed that the response of the graphene sensor was enhanced for the defective graphene layer. A gas sensor designed based on carbon nanotubes was investigated, and it was operated by a Wacker oxidation mechanism [35]. Instead of incorporating a metal-like copper that bonds directly to the gas, they used a palladium metal catalyst that added oxygen to ethylene during the oxidation process. 

However, in this study, we are planning to fabricate defect-induced CNTs, where defect-induced CNTs are a promising sensing layer for gas sensing [36,37]. The CNTs are a promising candidate for obtaining more induced defects and more interstitial regions. Hence, they are suitable for obtaining a good response and better performance towards gas molecules. It is feasible that the CNT-based current sensor induced by the defect should show a reasonable response towards ethanol gas. The novelty in the current work is that we do not use the mixing of any foreign elements after the fabrication of CNTs to obtain defect areas. Rather, the tubes were made with self-defects, which causes the presence of many defect bonds on the surface of the tubes, thus increasing the intrinsic sensitivity of the tubes towards gas molecules. Thus, we have fabricated carbon nanotubes (CNTs) using the chemical evaporation technique using 2 layers of nickel and chromium, where the thickness of the chromium layer was 5 nm, while the thickness of the nickel layers was 18 and 24 nm. The CNTs were prepared in the form of multi-walled nanotubes to have more defects induced inside them. We then studied this product using Raman spectroscopic, scanning, and transmission microscopy, and monitored the ethanol gas at various temperatures and gas concentrations. This product has been used to fabricate MWNT gas sensors by the drop-casting method for an ethanol gas, which has been tested at low temperatures and various concentrations. The possible gas sensing mechanisms are considered to visualize the understanding of the gas molecules’ interactions with the CNTs’ surface.

## 2. Materials and Methods

### 2.1. Materials

The substrate applied for the synthesis of the CNTs was SiO_2_/Si. During the fabrication of the catalyst layer with the dc-sputtering method, Ni and Cr layers (Labtech International Ltd., East Sussex, UK) were used as target materials. Nitrogen (99.995%), hydrogen (99.5%), and ethylene (>99.9%), provided by Air liquid LLC (Al Khobar, Saudi Arabia) were used as synthesis gases. High-purity synthetic air (N_2_ + O_2_) and diluted gases (NO, NO_2_, and CO) were provided for sensing measurements.

### 2.2. Methods

**Figure 1:**
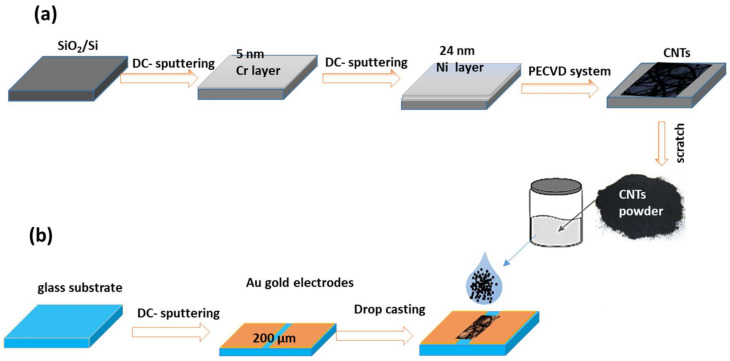
Schematic diagram for; (**a**) CNTs’ synthesis, and (**b**) sensing device fabrication.

For the synthesis of the CNTs, the polished SiO_2_/Si substrate was cleaned by sequence steps with water, Ethanol, and semiconductor detergent, then was dried with N₂. A Cr layer of 5 nm followed by an 18 nm and 24 nm layer of Ni was deposited by dc-sputtering. This catalyst layer was processed to plasma-enhanced chemical vapor deposition for the CNTs’ fabrication. The growth was carried out by using a fully automatic system called the Firstnano model-3000-Easy tube system. The growth temperature was 800 °C by a flow of the mixture of ethylene and nitrogen gases. The reaction time was 30 min, then the system goes down to room temperature; the steps are described in Figure 1. 

The product of carbon materials was firstly investigated by a confocal Raman Spectroscopy (Model: LabRAM- HR800, HORIBA, Kyoto, Japan) with an attached charge-coupled detector (CCD). The excitation laser HeNe with a wavelength of 633 nm and 20 mW for output power was used. The A backscattering configuration at room temperature with a 0.8 cm^−1^ spectral resolution was used for Raman spectrum measurements. The morphology was observed by an FE-SEM, Model JEOL JMS-7000 (Akishima, Japan) operating at 15 kV. The lattice image was examined by a JEOL JEM-2100F working at 200 kV.

**Figure 2:**
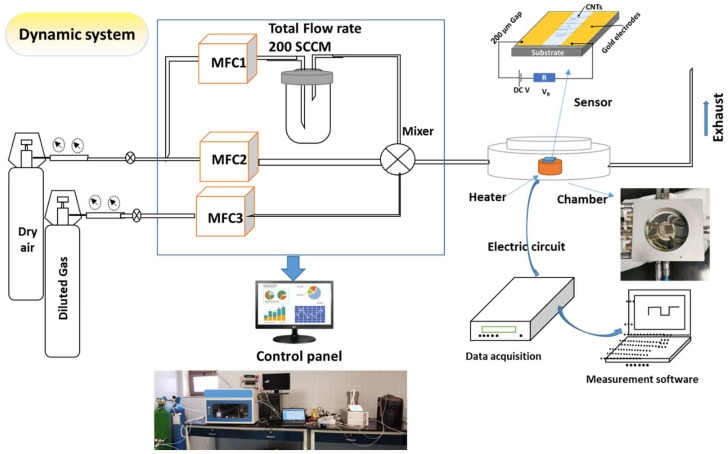
Schematic diagram of the gas sensing system.

The black layer powder formed on the substrate was scratched and dissolved in ethanol liquid. A few micro letters were deposited into the Au electrode with a gap of 200 μm. The Au electrodes were deposited by dc-sputtering. The sensing device was tested for various gases, such as NO, CO, acetone, NO_2_, and ethanol. The device was tested at different operating temperatures and gas concentrations of ethanol gas. The sensing device was dried at 70 °C before starting the sensing measurements. The measurements were performed with a dynamic gas system containing an electrical measurement system in a Keithley (Cleveland, OH, USA) meter and power supply, and a temperature-controlled chamber in the Linkam (Redhill, UK) model HFS 600 E—PB 4 probe thermal stage, and a gas flow control system of the GSM—6000 A model, as shown in Figure 2. The total flow rate of air and gas-containing air was adjusted for 200 SCCM for all experiments. The electrical current was measured by using a data acquisition of Keithley-2010 and data recorded by SweepMe! 1.5.5 software. The sensor response (*S*%) was calculated according to $S\%=\frac{R_{g}-R_{a}}{R_{a}}\times 100$, where *R_a_* and *R_g_* are the electrical resistance of the device in air and air-containing gas, respectively. 

## 3. Results

### 3.1. Structure Characterizations

**Figure 3:**
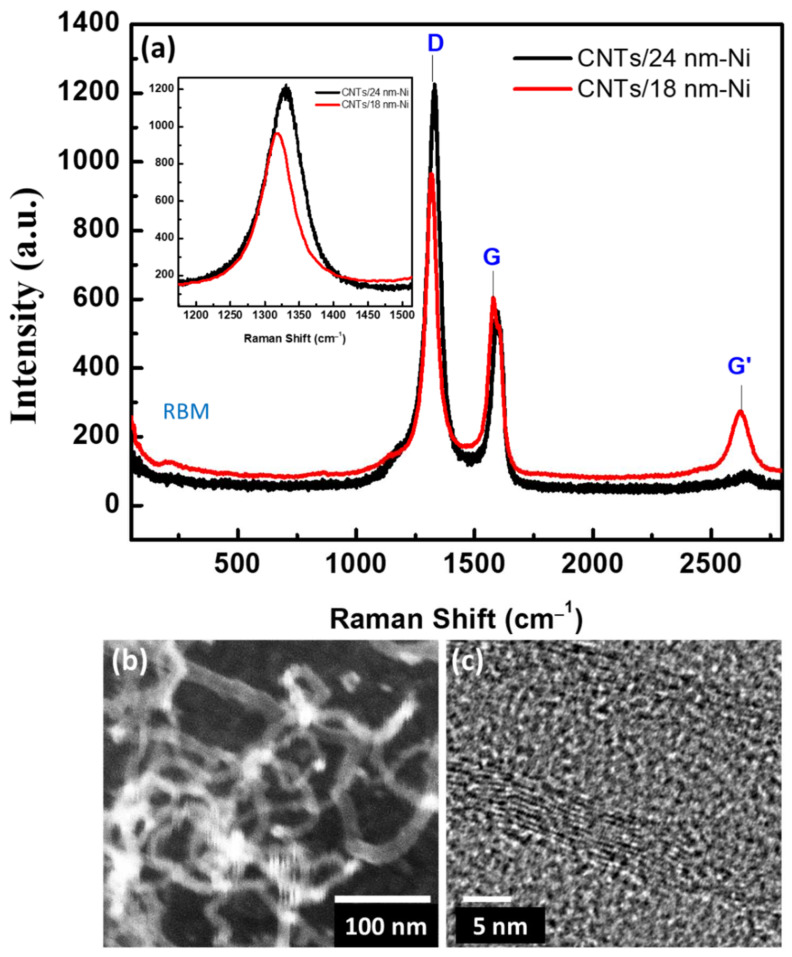
(**a**) Raman spectrum of the CNTs prepared with 18 and 24 nm of Ni catalyst layers, (**b**) FESEM image, and (**c**) HRTEM image CNTs prepared with 24 nm of Ni catalyst layers.

Raman spectroscopy is a sensitive tool for the disorder and the degrees of crystallinity for the materials [38,39]. The CNTs’ defects can be qualitatively characterized by Raman measurements. CNTs as a carbon material demonstrate three important modes described according to their surface defects, impurity, and layer multiplications, as shown in Figure 3a. These three modes were assigned to the D-band, G-bound, and G’-band, respectively. Other bands appearing at the low energy part of the spectrum are known as radial breathing mode (RBM) bands. This RBM band is distinctive for SWNTs and corresponds to the expansion and contraction of the tubes. The frequency of this band is correlated to the diameter of SWNTs. MWNTs have comparable spectra to SWCNTs. The difference is the lack of an RBM mode in MWNTs and a more pronounced D band in MWNTs. The RBM model is not present for the reason that the outer layers inhibit the breathing modes. The pronounced D band in MWNTs is due to a particular level because of the multilayer configuration and indicates further disorder in the structure. From Figure 3a, the D-band is observed to be higher for CNTs/24 nm-Ni compared to that for CNTs/18 nm-Ni. The band position of the D and G modes is listed in Table 1, corresponding to the ratio in intensities for both samples. In the present CNTs, the ratio of D/G intensities is 1.58 and 2.21 for CNTs/18 nm-Ni and CNTs/24 nm-Ni, expressing the more defect-induced CNTs/24 nm-Ni [40]. The G-bond is assigned to the intra-bond of sp^2^ pairs of hybridized carbons combined with E_2g_ symmetry, while the D-band assigns to the A_1g_ symmetry of the breathing mode owing to the disordering [41]. The ratio of G′ to G intensities are 0.46 and 0.16 for CNTs/18 nm-Ni and CNTs/24 nm-Ni, indicating the multilayer carbons for CNTs/24 nm-Ni compared to CNTs/18 nm-Ni [42,43,44]. Moreover, the RBM model appears very weak to CNTs/18 nm-Ni, which may confirm that there are few SWCNTs. 

The synthesized product was characterized by FWESEM, as shown in Figure 3b. The nanotubes were well-formed based on the catalyst layers and showed porous and curved nanotubes with a diameter of 11–12 nm. The FESEM image exhibits a crossed carbon nanotube. The observed nanotubes are porous structures and are interconnected with each other. HRTEM images also demonstrated well the size of the nanotubes and their crystallinity, as seen in Figure 3c. The HRTEM demonstrated that the CNTs have a multi-layer since the diameter is about 11 nm, as observed in the SEM image. This type of interconnected and porous structure is fitted with the gas sensing properties for the adsorption/desorption process, where the microstructures of materials have active sites due to the defects. Thus, there is a high probability for gas reactions on the surface of the CNT layer. Thus, the present CNTs have many surface defects which work as active adsorption sites. 

The measured surface area of the as-prepared MWNTs is in the range of 10 to 500 m^2^/g [45]. However, the theoretical external surface area of CNTs has been reported to be in the range of 50 to 1315 m^2^/g [46]. The theoretical specific surface area (SSA) of MWCNTs was calculated in terms of the external diameter and the number of layers of CNTs by Equation (1):

(1) $$\mathrm{SSA}\left(\mathrm{MWNT}\right)=\frac{1315\;de}{nde-0.68\sum_{i=1}^{n-1}i\;}$$

where *de* is the external diameter of the CNT, and *n* is the number of layers/shells that comprise the CNT. The specific surface area of the current CNTs/24 nm-Ni calculated via the above equation was ~150 m^2^/g, where *de* is 11.0 nm, and *n* is ~14.0 layers based on the fact that the inner diameter of the first tube is 2.0 nm (as for SWCNT) [45] and the inner-shell distance, *ds-s*, is 0.34 nm. 

### 3.2. Sensing Characterizations

**Figure 4:**
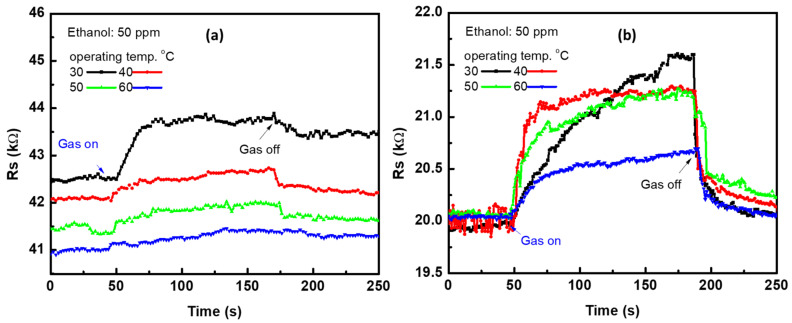
Single sensor signal at various operating temperatures ranging from 30–60 °C for CNTs prepared with (**a**) 8 nm and (**b**) 24 nm of Ni layer.

A gas sensor was fabricated by using the CNTs prepared using 18 nm and 24 nm of Ni layer. The sensor was tested at a temperature range of 30–60 °C. Figure 4 shows the signal of one cycle of 50 ppm of ethanol at temperatures of 30, 40, 50, and 60 °C. Figure 4a shows the signal of CNTs prepared with 18 nm of Ni catalyst layer, whereas Figure 4b shows that for CNTs prepared with 24 nm of Ni layer. The electrical resistance of the sensor increased due to the exposure to the gas, reaching up to its maximum state, and then decreased when the gas flow was switched off. It seems that the ethanol molecules inject electrons at the effective sites of CNTs’ surface, then the electrons are released back when the gas was stopped. This caused an increase and then decrease in the electrical resistance of p-type CNTs [47,48,49]. With increases in the operating temperature, the electrical resistance almost did not change significantly in the air. However, a notable change was observed in the presence of the gas, where the electrical resistance in the presence of gas molecules decreased with increases in the operating temperature from 30 up to 60 °C. From Figure 3, we can observe the large change in the sensor resistance prepared with 24 nm Ni compared to 18 nm. Moreover, the resistance value in the air was observed to be higher for CNTs prepared with 18 nm of Ni catalyst layer. This may be ascribed to the low defects in this sample, which was confirmed by Raman spectra. 

**Figure 5:**
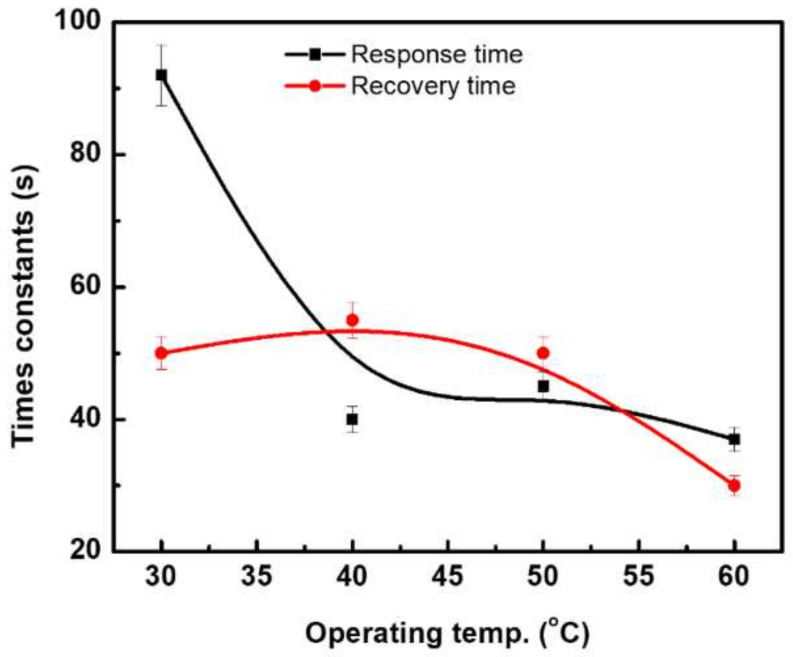
Response and recovery time constants at various operating temperatures for CNTs prepared with 24 nm of Ni layer.

The response time constant is defined as the time taken for the sensor resistance to reach 90% of the steady or equilibrium resistance, while the recovery time constant is the time taken for the sensor resistance to recover 90% of the steady resistance, or a 10% increment from the initial resistance. Figure 5 shows the response and recovery time constants for the sensor of CNTs/24 nm Ni (where it is the highly sensitive sensor) at various operating temperatures. The time constants were observed to decrease with increases in the operating temperature. The response time at the low operating temperature, where the high response was observed, is about 92 s, which was reduced to 37 s at an operating temperature of 60 °C, where the lower response was observed. The recovery time constant exhibited the same behavior depending on the sensor temperature. 

**Figure 6:**
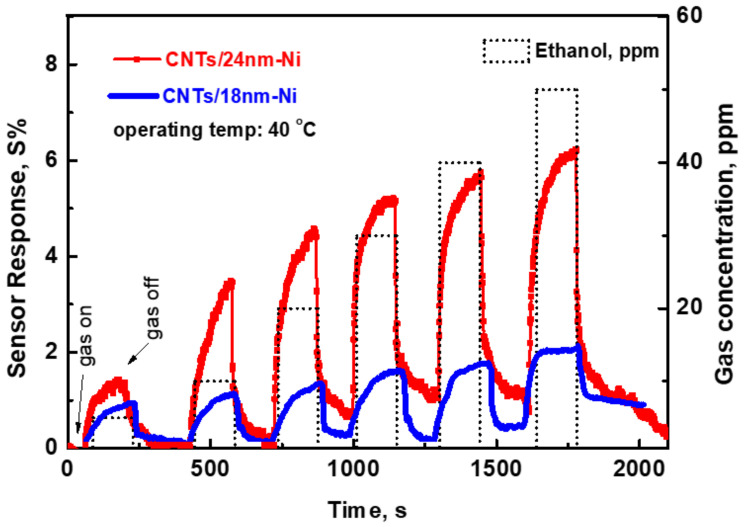
Sensor signal versus time at various gas concentrations for the sensor prepared with 18 nm and 24 nm of Ni catalyst layer.

Figure 6 depicted the change in the sensor response upon exposure to different gas concentrations for sensors prepared with CNTs/24 nm Ni (as an example). The sensor’s response toward various ethanol concentrations was investigated to understand the sensor’s capability to distinguish the gas concentration. The concentration was changed from 5 up to 50 ppm at an operating temperature of 40 °C. The sensor exhibits its ability to detect a lower concentration of ethanol. The sensor responded and recovered back to a proximate baseline periodically. With increases in the concentration, the recovery may take more time than that of low concentrations. This can be ascribed to the deep diffusion of gas molecules into the sensing layer, which causes slow desorption of these molecules. 

**Figure 7:**
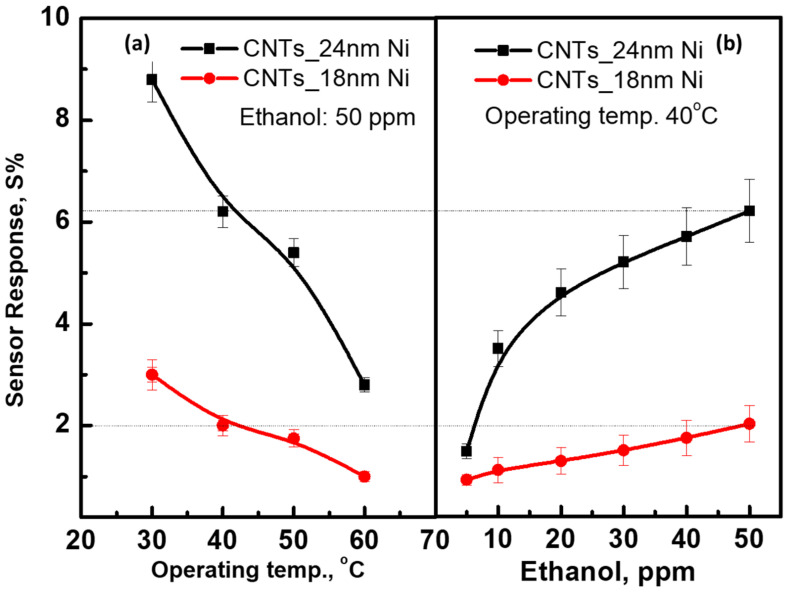
The sensor response of the prepared device is a function of; (**a**) operating temperatures at 50 ppm and (**b**) gas concentration at an operating temperature of 40 °C.

Figure 7 depicts very important curves for the temperature and concentration dependence of the sensor-response-prepared CNTs using 18 and 24 nm of Ni catalyst layers. The sensor response was recorded with a high value of 8.8% at an operating temperature of 30 °C (~RT) and a gas concentration of 50 ppm for the CNTs prepared with 24 nm of Ni layer, while the maximum response for the CNTs sensor prepared with 18 nm of Ni layer was 3.0%. Then, it decreased gradually to 6.5, 5.5, and 2.8% at 40, 50, and 60 °C for CNTs/24 nm-Ni sensors, respectively, and to 1.0% for CNT/18 nm-Ni. The result shows the sensor is more sensitive at a low temperature of 30 °C, which is considered important in saving power consumption. The sensor response of the CNTs/24 nm-Ni layer exhibited its superiority over the sensor prepared by the CNTs/18 nm-Ni layer. To understand the reduction in the sensor response with increases in the temperatures, we may propose the study of Albesa et al. [50]. The study was of the adsorption isothermal behavior of the ethylene gas on the surface of CNTs. The initial adsorption of gas began at very low pressures at the inner active sites of the nanotubes. The study was performed at 153, 273, and 343 K. At 153 K, the first layer of gas molecules on the outer surface of the carbon nanotubes was formed. For all three temperatures, the density of the adsorbed particles at the inner sites of the tubes was almost unchanged, but the instability of the gas particles layer was observed on the outer surface with increases in the temperature. This reflects that the adsorption of gas molecules is expected to be less onto the outer surface at higher temperatures. This explains the low response of the sensor at high temperatures. Figure 7b shows the calibration curve, describing the sensor response versus the gas concentration. The response was recorded at 1.5% and 1.0% at 5 ppm and increased up to 6% and 3.0% at 50 ppm for the sensors fabricated by CNTs/24 nm-Ni and CNTs/18 nm-Ni, respectively. The low rate of the sensor response with higher concentrations can be ascribed to the completion of the first layer of gas molecules on the surface, occupying most of the active sites of the outer surface. However, inserting more concentrated gas causes more molecules to reach the deep active sites of the CNTs, resulting in a gradual increment in the response.

**Figure 8:**
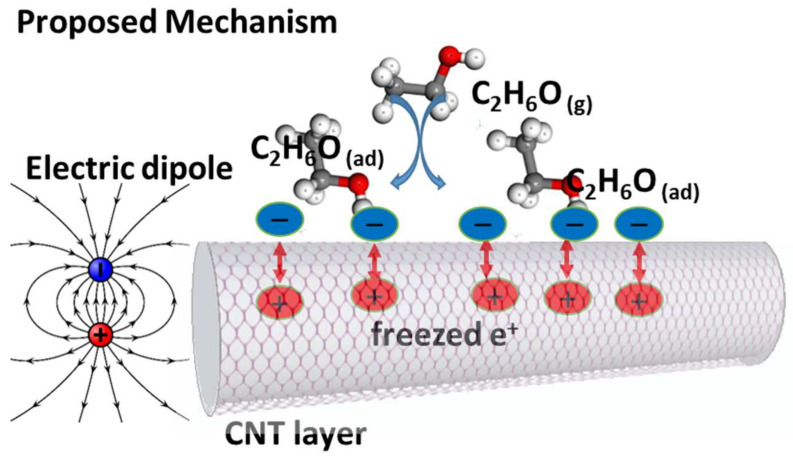
Proposed gas sensing mechanism based on Coulomb’s interaction.

Works of literature have reported the p-doping type of CNTs [47,48,49]. There are two types of sensing mechanisms that can describe the behavior of the sensor response to the analyte. First is the intra-CNTs mechanism, which describes the interaction between CNTs and analytes [51]. The second is the contact mechanism, which describes the effect of contact electrodes. In the former mechanism, the negative charge is transferred from the Au electrode to the CNT, resulting in the equilibrium of the Fermi level of both the materials [52,53]. When the ethanol molecules reach the surface, they may be linked at the contacts, preventing the transport of electrons to the CNTs. However, because of the p-type of CNTs, the resistance should decrease due to more positive charges becoming free, which was not in the present case. Moreover, the contributing possibility of this mechanism to the sensor conductivity is low due to the small area of the contacts. Thus, we can say that the Schottky barrier effect between Au contacts and CNTs can be ignored. In the latter mechanism when CNTs are exposed to a reducing gas, the positive charge density decreases flowed to an increase in the electrical resistance, while the oxidizing gas showed the reverse effect. The ethanol molecules find their way to the interstitial and defective points of the CNT’s surface. Thus, the direct interaction of the charge transfer was considered the main sensing mechanism [51]. Furthermore, we can also propose another suitable mechanism based on the Coulomb interaction force. In its nature, ethanol is comprised of polar molecules because of the hydroxyl group (-OH) connected to the carbon end, where the electronegativity of oxygen and hydrogen is 3.44 and 2.2, respectively. This difference in the electronegativity of hydrogen and oxygen atom causes the polarity of the hydroxyl group, resulting in a non-zero dipole moment of ethanol molecules that make it polar. Thus, the physical adsorption of the ethanol molecules is enough to create a Coulomb force (electric dipole) between the p-CNTs and n-ethanol, creating a depletion layer, as schemed in Figure 8. This type of electric dipole causes an increase in the sensor resistance due to the freezing of the holes in the CNTs. 

**Figure 9:**
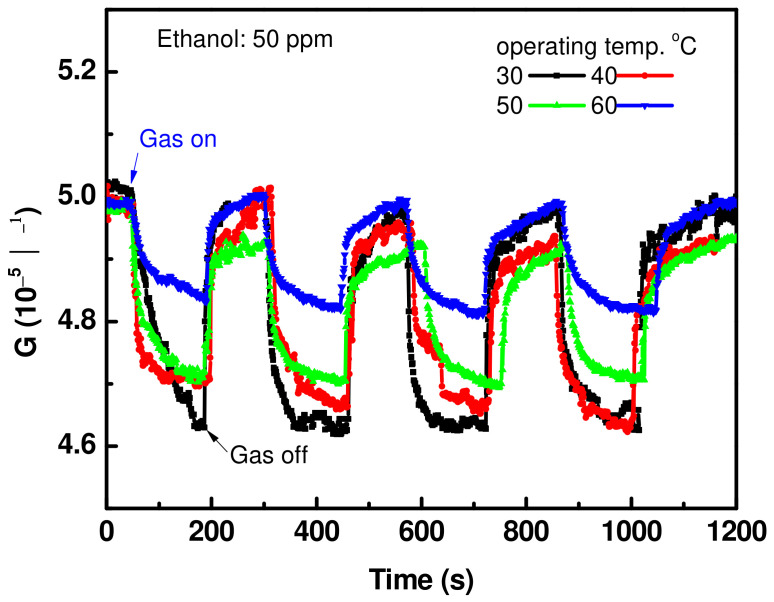
Cyclic curve of sensor signals at concentrations of 50 ppm, and various temperatures for the sensor fabricated with CNTs/24 nm-Ni layer.

**Figure 10:**
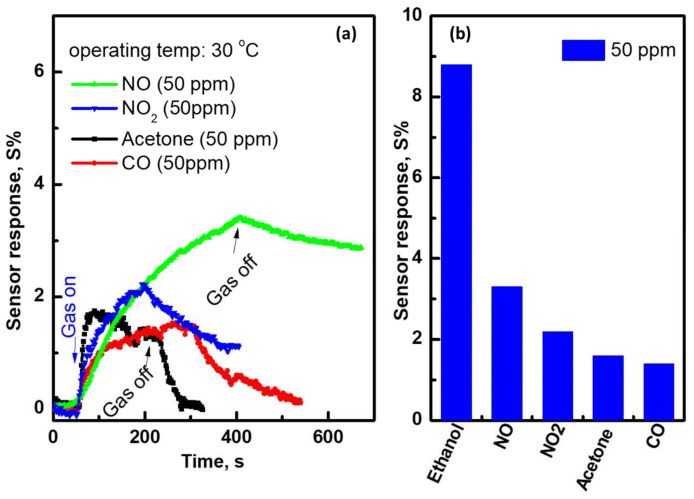
(**a**) Sensor signal toward various gases: NO, NO_2_, acetone, and CO, and (**b**) the sensor’s selectivity toward these gases compared to ethanol measured at 30 °C, for the sensor fabricated with CNTs/24 nm-Ni layer.

The repeatability verification, which indicates the repeated output signal of the sensor signal, is a very important parameter for understanding the sensor’s ability with regard to the change in environment. The sensor’s conductance was measured periodically for four cycles for the gas concentration of 50 ppm at various operating temperatures, as shown in Figure 9. This figure shows the change in sensor conductance, G, as a function of time in terms of the existence and exhaustion of the ethanol gas. The sensor responded quickly to the adsorbed gas molecules and recovered back to the baseline when the gas was switched off. At low operating temperatures, the sensor shows a large change in its electrical conductance, while at higher temperatures, a small change is observed. The sensor repeatedly responded to the gas molecules without observing drift in the cycles, which confirms the ability to repeat its outputs without delay. For deep sensing investigations, the sensor was tested at the highest sensor response temperature for different gases. Figure 10a shows the sensor response toward reducing and oxidizing gases such as NO, NO_2_, Acetone, and CO. The sensing measurements were carried out under the same conditions, which means 50 ppm and an operating temperature of 30 °C. A quick response and recovery were observed for CO and acetone compared to NO and NO_2_, but with a low response. Figure 10b expresses the selectivity towards ethanol compared to others. The results confirmed that the current fabricated CNT’s sensing layer is superior for detecting ethanol gas at low operating temperatures and low concentrations, as well. Table 2 presents the sensor response and operating temperature toward ethanol gas for various sensors fabricated based on the CNTs layer. The data presented here expressed its superiority in terms of sensing parameters. 

**Figure 11:**
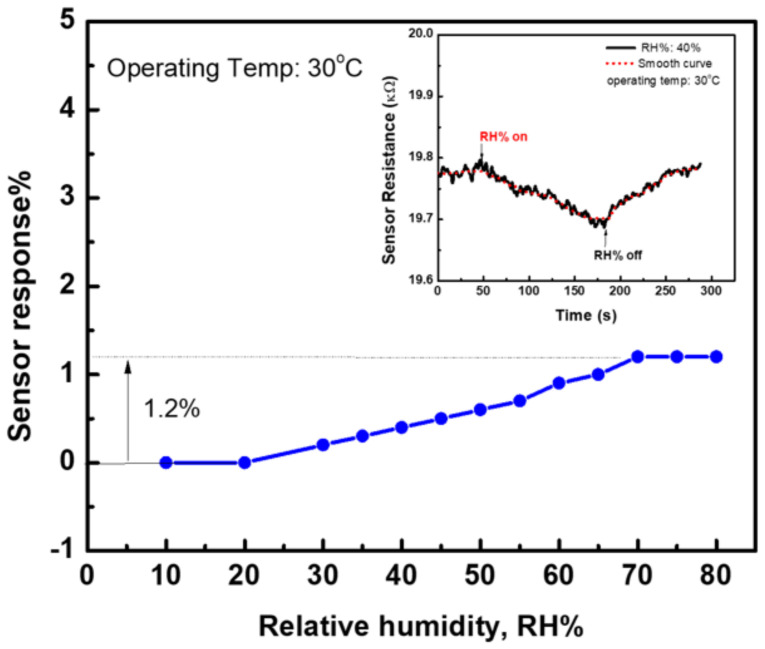
The sensor response at an operating temperature of 30 °C for various humidity conditions for the sensor fabricated with CNTs/24 nm-Ni layer.

Figure 11 shows the sensor response at an operating temperature of 30 °C towards various humidity conditions (Humidity Series, Sheldon Manufacturing, Inc., Cornelius, OR, USA). The sensor response shows no change at humidity levels of 10–20%Rh, but a slight change in the sensor response at higher humidity values. The sensor showed a response with a value of 1.2% when the humidity level increased up to 70%Rh. The inset figure demonstrated the change in sensor resistance at a humidity level of 40% Rh. The resistance decreased when the sensor was exposed to the high humidity level, which is opposite to the behavior of the resistance when the sensor was exposed to ethanol gas, although the effect of humidity is opposite to the effect of ethanol gas. Thus, the observed results confirm the ability of the present sensor to work well at various levels of humidity conditions. 

## 4. Conclusions

In summary, highly defective multiwalled carbon nanotubes were successfully synthesized by chemical vapor deposition based on 24/5 nm of Ni/Cr as a catalyst layer for highly sensitive ethanol gas. The sensor prepared with CNTs on the 24 nm-Ni catalyst layer exhibited its superiority compared to that prepared with CNTs on the 18 nm-Ni catalyst layer. The growth of CNTs was confirmed by observing the breath and stretching modes of the Raman spectrum, as well as by FESEM and HRTEM. The CNTs were used to fabricate a sensing device which was examined for its efficacy towards various types of gas, showing the selectivity towards ethanol gas. Then, the ethanol sensing properties were systematically investigated at different operation temperatures and gas concentrations. The highest response of 8.8% was observed at 30 °C and 50 ppm concentrations. The response decreased to 2.8% with increases in the temperature of up to 60 °C. Moreover, the sensor exhibited its capability for detecting various ethanol concentrations. The gas sensing mechanism of direct interaction between the CNTs’ defect points and gas molecules was considered as the main mechanism here. We also proposed another mechanism based on physical adsorption, which we think can be a possible mechanism for such cases.

## Figures and Tables

**Table 1:** D- and G-band position, and the intensities ratios of D/G and G’/G peaks.

Sample	D-Band (cm^−1^)	G’-Band (cm^−1^)	I_D/G_	I_G’/G_
CNTs/18 nm-Ni	1317	1565	1.58	0.46
CNTs/24 nm-Ni	1330	1595	2.21	0.16

**Table 2:** The ethanol sensor response and operating temperature based on published CNT work.

Sensor	Operation Temp. (°C)	Response	Ethanol Concen. (ppm)	References
High-density CNTs	RT	0.18 ^b^	50	[54]
MWNTs/ZnO	250	45 ^a^	50	[23]
MWNT film devices	RT	4.1 ^b^	30,000	[28]
MWCNT	RT	3.0 ^b^	50	[55]
PEG/MWCNTs	RT	2.9 ^b^	50	
0.05 wt% MWCNT/SnO_2_	250	7000 ^a^	300	[56]
0.10 wt% MWCNT/SnO_2_	250	1900 ^a^	300	
MWCNTs	RT	8.8 ^b^	50	This work

^a^: response = $\frac{R_{a}}{R_{g}}$ ^b^: response = $\frac{\Delta R}{R}\times 100$.

## Data Availability

Based on request.
